# Trajectories of PrEP use among men who have sex with men: a pooled analysis of two prospective, observational cohort studies

**DOI:** 10.1002/jia2.26133

**Published:** 2023-07-27

**Authors:** Vita W. Jongen, Thijs Reyniers, Maarten Schim van der Loeff, Tom Smekens, Elske Hoornenborg, Mark van den Elshout, Hanne Zimmermann, Liza Coyer, Chris Kenyon, Irith De Baetselier, Udi Davidovich, Henry J. C. de Vries, Maria Prins, Marie Laga, Bea Vuylsteke, Anders Boyd

**Affiliations:** ^1^ Department of Infectious Diseases Public Health Service Amsterdam Amsterdam The Netherlands; ^2^ Department of Public Health Institute of Tropical Medicine Antwerp Belgium; ^3^ Department of Internal Medicine University of Amsterdam Amsterdam The Netherlands; ^4^ Amsterdam Institute for Infection and Immunity (AII) Amsterdam The Netherlands; ^5^ Amsterdam Public Health Research Institute (APH) Amsterdam The Netherlands; ^6^ Department of Work and Social Psychology Maastricht University Maastricht The Netherlands; ^7^ Department of Clinical Sciences Institute of Tropical Medicine Antwerp Belgium; ^8^ Department of Social Psychology University of Amsterdam Amsterdam The Netherlands; ^9^ Department of Dermatology University of Amsterdam Amsterdam The Netherlands; ^10^ Stichting HIV Monitoring Amsterdam The Netherlands

**Keywords:** cohort studies, Europe, HIV prevention and control, men who have sex with men, pre‐exposure prophylaxis, public health

## Abstract

**Introduction:**

Daily and event‐driven oral pre‐exposure prophylaxis (PrEP) reduce the risk of HIV acquisition. PrEP use can vary over time, yet little is known about the trajectories of PrEP use irrespective of the chosen PrEP regimens among men who have sex with men (MSM).

**Methods:**

Using data from a mobile, web‐based diary application collected daily from 17 August 2015 until 6 May 2018, we analysed PrEP use and sexual behaviour in two large cohorts, AMPrEP (Amsterdam, the Netherlands) and Be‐PrEP‐ared (Antwerp, Belgium). In both cohorts, participants could choose between daily and event‐driven oral PrEP every 3 months. We used group‐based trajectory modelling to identify trajectories of PrEP use over time and their determinants. In addition, we estimated the incidence rate of chlamydia, gonorrhoea and syphilis within these trajectories.

**Results:**

We included 516 MSM (*n* = 322 AMPrEP; *n* = 194 Be‐PrEP‐ared), of whom 24% chose event‐driven PrEP at PrEP initiation. Participants contributed 225,015 days of follow‐up (median = 508 days [IQR = 429−511]). Four distinct PrEP use trajectories were identified: ≤2 tablets per week (“low frequency,” 12% of the total population), 4 tablets per week (“variable,” 17%), “almost daily” (31%) and “always daily” (41%). Compared to participants with “low frequency” PrEP use, participants with “variable” (odds ratio [OR] = 2.18, 95% confidence interval [CI] = 1.04−4.60) and “almost daily” PrEP use were more often AMPrEP participants (OR = 2.64, 95% CI = 1.27−5.49). “Almost daily” PrEP users were more often employed (OR = 6.76, 95% CI = 2.10−21.75) and were younger compared to participants with “low frequency” PrEP use. In addition, the number of days on which anal sex occurred was lower among participants with “low frequency” PrEP use compared to the other groups (all *p*<0.001). Compared to “low frequency” PrEP users, the incidence rates of chlamydia and gonorrhoea were higher for participants with “almost daily” and “always daily” PrEP use.

**Conclusions:**

We uncovered four distinct PrEP use trajectories, pointing to different patterns of PrEP use in practice beyond the two‐regimen dichotomy. These trajectories were related to sexual behaviour and rates of sexually transmitted infection. Tailoring PrEP care according to different PrEP use patterns could be an important strategy to improve efficient PrEP delivery.

## 1 INTRODUCTION

Oral pre‐exposure prophylaxis (PrEP) is highly effective in reducing the risk of HIV acquisition when taken correctly [1, 2, 3]. Among cisgender men who have sex with men (MSM), PrEP can be taken as a daily or event‐driven regimen [4, 5]. Event‐driven PrEP (also referred to as “intermittent,” “on‐demand” or 2‐1‐1 PrEP) involves taking two tablets 24–2 hours before an anticipated sex act, followed by a daily tablet every 24 hours until 48 hours after the last sex act [4].

For daily PrEP, variation in use is limited to stopping and restarting a daily PrEP regimen. For event‐driven PrEP, there has been a large variation observed in the use of this regimen [6]. The Be‐PrEP‐ared (Antwerp, Belgium) and Amsterdam PrEP (AMPrEP) (Amsterdam, the Netherlands) cohort studies were the first demonstration projects worldwide where participants could choose and switch between daily and event‐driven regimens during follow‐up [7, 8]. We previously showed that about one‐third of participants switched regimens at least once within 28 months after initiating PrEP [9]. Some participants also discontinued daily PrEP for prolonged periods of time before restarting this regimen again [10, 11]. An important insight from these studies is that, in practice, PrEP use is often adapted to changing needs [10, 12, 13], and defining PrEP use as either daily or event‐driven might not appropriately encompass the behaviours and risk of acquiring sexually transmitted infections (STIs) and HIV.

Identifying more refined patterns of use, and what types of behaviours and outcomes are associated with these patterns, may help inform PrEP programmes by allowing more tailored care and more efficient PrEP delivery. In this study, we established PrEP use trajectories and their determinants among MSM participating in the Be‐PrEP‐ared and AMPrEP studies. In addition, we assessed the incidence rate (IR) of syphilis, gonorrhoea and chlamydia within these trajectories.

## 2 METHODS

### 2.1 Study design and participants

For this study, we pooled pseudonymized data from the Be‐PrEP‐ared (2015–2018) and AMPrEP (2015–2020) cohort studies. Full procedures of both studies have been published previously [7, 8]. Briefly, HIV‐negative MSM and transgender women were eligible for participation if they were at least 18 years of age and reported any one of the following in the 6 months before enrolment: (1) condomless anal sex with casual partners; (2) at least one diagnosed (bacterial) STI; (3) post‐exposure prophylaxis (PEP) use; or (4) sex with a partner living with HIV and with an unknown or detectable viral load (AMPrEP only). As only a few transgender women (*n* = 5) were included in both studies, they were excluded from these analyses.

At PrEP initiation, participants could choose between daily and event‐driven PrEP, and could switch between regimens at every 3‐monthly study visit. Study staff explained both regimens. During follow‐up visits, these regimens were sometimes discussed, depending on the needs of the participant (e.g. when preferring or hesitating to switch; or when assessing adherence).

Since follow‐up for the Be‐PrEP‐ared study was shorter than for AMPrEP, we included data for the first 73 weeks after PrEP initiation for all participants (i.e. the time at which the majority of Be‐PrEP‐ared participants reported daily data). The study period for this analysis included visits from 17 August 2015 until 6 May 2018.

### 2.2 Study procedures

For the Be‐PrEP‐ared study, a personal web‐based diary application (app), and later an additional smartphone app, were used to collect daily information on PrEP use. For the AMPrEP study, an app was used to collect daily information on PrEP use [14, 15]. On each day, Be‐PrEP‐ared participants indicated whether PrEP was used and how many tablets (ranging from 1 to 3), whereas in AMPrEP, participants only indicated whether or not PrEP was taken on a given day. Participants could also indicate whether they had anal sex on that day. During study visits, Be‐PrEP‐ared participants were asked to retrospectively complete missing data in the web‐based diary. AMPrEP participants were not actively reminded to fill in the diary. At PrEP initiation and 3‐monthly study visits, participants completed questionnaires on socio‐demographics (e.g. age) and sexual behaviour (e.g. number of sex partners).

Participants were screened for syphilis, chlamydia and gonorrhoea at 3‐monthly study visits [10, 11]. Results from additional STI testing between study visits were included in the analysis.

### 2.3 Statistical analysis

To exclude participants with overly unreliable data, we included those who reported data on PrEP use for at least 10% of all days during the entire 73‐week follow‐up period after the date of PrEP initiation (i.e. roughly the fifth percentile of % reporting data). To assess potential bias from non‐response, we compared the distribution of baseline socio‐demographic and sexual behaviour variables of participants who reported data in the apps at least 10% of days during follow‐up to those who did not.

Follow‐up started at PrEP initiation (i.e. baseline) and continued until PrEP discontinuation (as indicated by the participant) or 73 weeks after PrEP initiation, whichever occurred first.

We used group‐based trajectory models to identify groups of participants who followed distinct individual‐level trajectories of PrEP use [16]. Group‐based trajectory models are a form of finite‐mixture models that use a multinomial modelling strategy to identify clusters of trajectories within a study population. We modelled trajectories as a cubic function of time using the Stata “traj” plug‐in. We fitted a model with a censored normal distribution using the number of days of PrEP use per week (range 0–7) as the outcome. We made no distinction between one or two tablets taken on a given day. If information on PrEP use was available at least once during a given week, missing data of that week were considered as not taking PrEP. If information on PrEP use was available for none of the days during a given week, the entire week was considered missing and these data were not included in the model.

We ran a series of group‐based trajectory models with increasing numbers of groups (i.e. *k* = 1, 2, …, 6) and determined the optimal number of groups from the model resulting in the lowest Bayesian Information Criteria, highest entropy values, and all trajectories having a marginal prevalence of >10% [16]. Based on this procedure, a total of four distinct trajectories were identified (Table S1). Group‐based trajectories and observed group means of PrEP use per week were plotted using the “trajplot” command in Stata. The four‐group model was also stratified by study (i.e. AmPrEP or Be‐PrEP‐ared).

We assigned participants to a trajectory based on the highest a posteriori probability of group membership. We compared the distribution of baseline socio‐demographic and sexual behaviour variables between trajectory groups, as determined from the main model, using the Kruskal–Wallis test for continuous variables and Pearson's χ^2^ or Fisher's exact tests for categorical variables. We tested for interaction between each variable and study using an analysis of variance test.

As group membership is based on a finite‐mixture distribution (i.e. group membership contains some degree of misclassification), we modelled the probability of belonging to each group across several covariates directly in the group‐based trajectory models. Univariable odds ratios (OR) of time‐stable covariates associated with group membership, and their 95% confidence intervals (CI), were calculated from the main model. We constructed a multivariable model by adding covariates with a *p*<0.20 in univariable analysis. We removed covariates that were not significant in all groups from the multivariable model in a backward‐stepwise fashion. To account for differences between cities, we forced study as a covariate in the multivariable models. We also tested whether the effects of each covariate in the multivariable model were different between studies by including a covariate × study interaction term, separately, for each covariate.

To examine the association between group membership and STI incidence, we estimated chlamydia, gonorrhoea and syphilis (primary secondary and early latent) IRs per 100 person‐years by dividing the number of incident infections by person‐years of observation. Repeated infections over time were included in the analysis and all follow‐up time was considered time at risk. This analysis was stratified by PrEP use trajectory groups, as assigned by the highest a posteriori probability of membership. We estimated incidence rate ratios (IRRs) and their 95% CI, adjusted for age and STI testing frequency per total months in follow‐up, using Poisson regression to compare the relative differences in STI incidence between participants belonging to different trajectories. Again, we tested whether effects were different between studies by including a profile × study interaction term in the model.

Statistical analyses were performed using Stata (v15.1, StataCorp, College Station, TX, USA) or R (version 3.6.3, Vienna, Austria).

### 2.4 Ethical considerations

The Be‐PrEP‐ared study (EudraCT 2015‐000054‐37) was approved by the institutional review board of the Institute of Tropical Medicine, Antwerp (988/15), and the ethics committee of the Antwerp University Hospital (15/25/255). The AMPrEP study obtained ethical approval from the ethics board of the Academic Medical Center, Amsterdam, the Netherlands (NL49504.018.14). AMPrEP was registered at the online Dutch trial registry (NTR5411). All participants provided written informed consent.

## 3 RESULTS

Between August 2015 and December 2016, 571 MSM were included in the Be‐PrEP‐ared (*n* = 197, 35%) and AMPrEP (*n* = 374, 66%) studies. Of these participants, 516 (90%) reported data at least 10% of the days during the 73 weeks after PrEP initiation and were included in the analysis. Age did not differ between included and excluded participants (*p* = 0.207, Table S2). Excluded participants were more often AMPrEP participants (*p*<0.001), event‐driven PrEP users at baseline (*p* = 0.002) and unemployed (*p* = 0.005). Included participants more often had used PEP (*p* = 0.036) and more often were diagnosed with an STI in the 6 months before baseline (*p*<0.001).

When comparing participants from the Be‐PrEP‐ared and AMPrEP studies who were included in the analyses (Table S3), there were no significant differences in many of the socio‐demographic characteristics, with the exception of more individuals aged ≥45 years in the AmPrEP study (*p* = 0.002). Participants from Be‐PrEP‐ared also had a higher number of days completed in the app (*p*<0.001), higher proportion identifying as exclusively homosexual (*p* = 0.011), higher proportion of previous PEP use (*p* = 0.014) and STI at baseline (*p*<0.001) and higher number of anal sex days during follow‐up (*p*<0.001) (Table S3).

### 3.1 Trajectories of PrEP use

**Figure 1:**
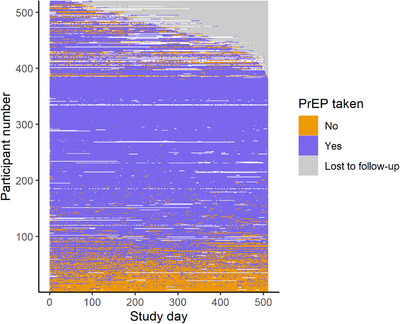
PrEP use per day during the first 73 weeks of study participation as recorded in the daily diary data. Be‐PrEPared and AMPrEP studies, 17 August 2015–5 May 2018. Note: Included are data of participants who entered data at least 10% of the days. Each row represents data from an individual participant over the course of follow‐up. Participants are stacked from bottom to top based loosely on the increasing proportion of PrEP use and then total follow‐up time. White squares represent days on which no data on PrEP use were provided.

**Figure 2:**
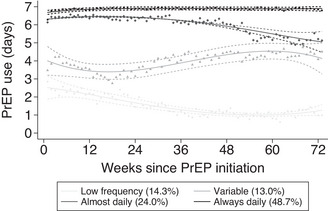
Trajectories of PrEP use per week over time among MSM and transgender PrEP users. Be‐PrEPared and AMPrEP studies, 17 August 2015–5 May 2018. Note: Plotted symbols indicate the observed group mean number of tablets per week within each trajectory; the plotted lines indicate the trajectory; dashed lines indicate the 95% confidence intervals of the trajectory.

**Figure 3:**
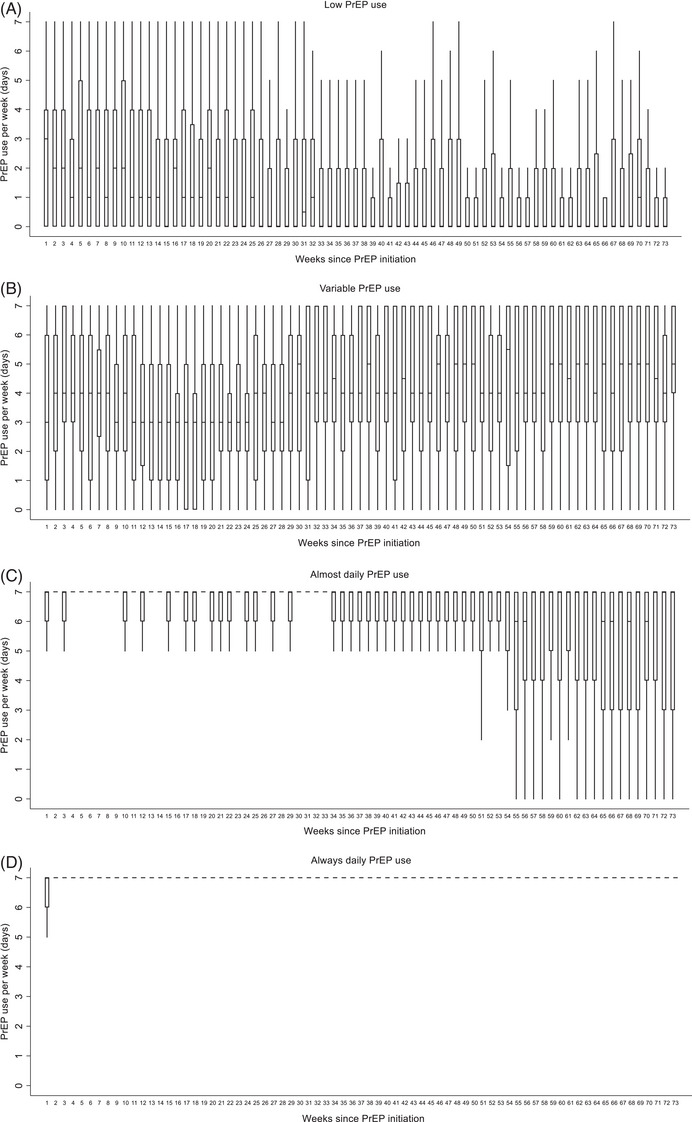
PrEP use per week during follow‐up within the different groups of PrEP use. Be‐PrEPared and AMPrEP studies, 17 August 2015–5 May 2018. Note: Box‐plots demonstrate the distribution of PrEP use per week, for every week during follow‐up, across the four PrEP use groups: low frequency (**A**), variable (**B**), almost daily (**C**) and always daily (**D**). The median is presented within the box, while the outer limits of the box represent the interquartile range. Whiskers extend to the minimum and maximum values. In the “almost daily” and “always daily” PrEP use groups, there were weeks for which the minimum and maximum values were the same (i.e. 7 days of PrEP use), while these weeks were represented as small strips in the figure. In some box‐plots, the 25th or 75thpercentile is the same as the median and is represented with a thicker strip.

Participants reported a total 225,015 days of data during 263,676 days of follow‐up, with a median of 508 days [IQR = 429−511]. PrEP use per day during the 73 weeks is visualized in Figure 1. Three hundred and twenty‐nine (64%) had at least 1 day on which they did not fill in the app. One hundred and thirty‐six (26%) had at least 1 entire week during which they did not fill in the app, with a median 8 [IQR 3−20] weeks considered as missing. Four distinct trajectories of PrEP use were identified (Figure 2). Approximately 12% of the 516 included participants used PrEP on average 2 days a week or less (termed herein as “low frequency”), 17% used on average 4 days per week with some variation around this average (“variable”), 31% used PrEP almost daily (“almost daily”) and 41% used PrEP always daily (“always daily”). The trajectories and their distributions were comparable between cities (Figures S1A and B); however, individuals from Be‐PrEP‐ared were rarely in the “almost daily” profile, which demonstrated a drastic decrease in PrEP use after week 48, possibly from few participants discontinuing PrEP (Figure S1B). Variation in weekly PrEP use was less visible in the trajectories with “always daily” or “almost daily” PrEP use than in the other two groups (Figure 3). Varying PrEP use can be seen for all trajectories, except for the “always daily” PrEP trajectory, and the “almost daily” PrEP use group appeared to show declines in number of days per week towards the end of follow‐up.

### 3.2 Determinants of PrEP trajectory membership

Participants with “always daily” PrEP use had the least missing data in the apps compared to the other trajectories (*p*<0.001, Table 1). Participants using PrEP “almost daily” were younger (*p* = 0.011). Participants with “low frequency” PrEP use were least often employed (*p* = 0.016) and had the lowest total number of reported anal sex days over the study period (*p*<0.001).

**Table 1:** Socio‐demographic characteristics and sexual behaviour^a^ according to PrEP use profile

Profile^b^										
	*Low frequency* *(n = 60)*		*Variable* *(n = 87)*		*Almost daily* *(n = 160)*		*Always daily* *(n = 209)*		*p‐value* *^c^* *variable only*	*p‐value* *^c^* *variable × study interaction*
	*n* ^d^	%^d^	*n* ^d^	%^d^	*n* ^d^	%^d^	*n* ^d^	%^d^		
**Number of days completed in the app** ^e^										
Median [IQR]	489	[225−511]	486	[205−510]	485	[342−510]	510	[507−511]	<0.001	0.917
**Study**									0.059	n.a.
Antwerp	27	45%	28	32%	50	31%	89	43%		
Amsterdam	33	55%	59	68%	110	69%	120	57%		
**Choice of PrEP regimen**									<0.001	0.174
Event‐driven	50	83%	52	60%	17	11%	4	2%		
Daily	10	17%	35	40%	143	89%	205	98%		
**Age (years)**										
Median [IQR]	40	[34−48]	40	[30−47]	36	[29−45]	40	[34−47]	0.011	0.968
< 35 years	18	30%	31	36%	67	42%	55	26%	0.097	0.907
35–44 years	21	35%	30	34%	49	31%	84	40%		
≥ 45 years	21	35%	26	30%	44	28%	70	33%		
**Self‐declared racial‐ethnic background**									0.981	0.569
White	52	87%	75	86%	138	86%	183	88%		
Non‐White	8	13%	12	14%	22	14%	26	12%		
**Highest education level**									0.572	0.538
No college/university	14	23%	14	16%	37	23%	46	22%		
College/university	46	77%	73	84%	121	77%	162	78%		
**Employment**									0.016	0.050
Unemployed	16	27%	15	17%	21	13%	22	11%		
Employed	44	73%	72	83%	139	87%	183	89%		
**Steady relationship**									0.069	0.880
No	34	57%	46	54%	101	63%	102	49%		
Yes	26	43%	39	46%	59	37%	105	51%		
**Living situation**									0.045	0.915
Alone	32	53%	43	49%	92	58%	98	47%		
With partner	20	33%	29	33%	37	23%	84	40%		
With others	8	13%	15	17%	31	19%	27	13%		
**Sexual identity**									0.169	0.838
Exclusively homosexual	59	98%	84	97%	150	94%	191	91%		
Not exclusively homosexual	1	2%	3	3%	9	6%	18	9%		
**CAS with casual partner** **^f^**									0.342	0.092
No	4	7%	3	3%	10	6%	6	3%		
Yes	56	93%	84	97%	150	94%	203	97%		
**Post‐exposure prophylaxis used** **^g^**									0.610	0.043
No	53	88%	79	91%	139	87%	190	91%		
Yes	7	12%	8	9%	21	13%	19	9%		
**Sexually transmitted infection** **^f^** **^,^** **^g^**									0.250	0.287
No	39	65%	44	51%	85	53%	106	51%		
Yes	21	35%	43	49%	75	47%	103	49%		
**Anal sex days** **^h^**										
Median [IQR]	46	[23−92]	96	[61−146]	106	[62−157]	159	[110−211]	<0.001	0.969

**Note: Be‐PrEP‐ared and AMPrEP studies, 17 August 2015–5 May 2018**.

**Abbreviations**: CAS, condomless anal sex; HIV, human immunodeficiency virus; IQR, interquartile range; n.a., not applicable; PEP, post‐exposure prophylaxis.

Data missing for: employment (*n* = 4), sexual identity (*n* = 1) and steady relationship (*n* = 4).

^a^ All determinants were determined at baseline, except for number of days completed in the app and number of anal sex days.

^b^ Participants were assigned to a group based on the highest posterior probability of belonging to a given class.

^c^ “*p*‐value variable only” compares distributions of each variable across profile groups using Pearson's χ^2^ or Fisher's Exact test for categorical variables and Kruskal–Wallis rank test for continuous variables. “*p*‐value variable × study interaction” tests for effect modification between variable and study using an analysis of variance test for interaction.

^d^ Unless otherwise indicated.

^e^ Participants could complete a maximum of 511 days in each of the applications.

^f^ In the 6 months before baseline.

^g^ At least one bacterial sexually transmitted infection (i.e. syphilis, or urethral or rectal chlamydia or gonorrhoea) at baseline.

^h^ Sum of the number of days on which anal sex occurred during follow‐up.

Univariable associations of determinants, when modelling the finite‐mixture distribution of trajectory membership in the group‐based trajectory model, are shown in Table S4. In the multivariable model (Table 2), we found that AMPrEP participants (*p* = 0.040) had higher odds of being “variable” PrEP users compared to “low frequency” PrEP users. Additionally, we found that AMPrEP participants (*p* = 0.009) and those employed (*p* = 0.001) had higher odds of being “almost daily” PrEP users compared to “low frequency” PrEP users, while older participants had lower odds of being “almost daily” PrEP users (age 35–44 years *p* = 0.044, age ≥45 years *p* = 0.008). Compared to “low frequency” PrEP users, participants in all other trajectories had more days on which they reported anal sex (all *p*<0.001). There was no evidence of interaction between any of the covariates and the study (Table 2).

**Table 2:** Multivariable determinants of PrEP use profiles

	Profile						*p*‐value^a^ covariable × study interaction
	Variable versus low		Almost daily versus low		Always daily versus low		
	*aOR (95% CI)*	*p‐value*	*aOR (95% CI)*	*p‐value*	*aOR (95% CI)*	*p‐value*	
**Study**							
Antwerp	REF		REF		REF		n.a.
Amsterdam	2.18 (1.04−4.60)	0.040	2.64 (1.27−5.49)	0.009	1.78 (0.97−3.28)	0.064	
**Age**							0.889
<35 years	REF		REF		REF		
35–44 years	0.78 (0.33−1.85)	0.568	0.44 (0.20−0.98)	0.044	0.79 (0.39−1.62)	0.523	
≥45 years	0.75 (0.32−1.77)	0.508	0.32 (0.14−0.74)	0.008	0.59 (0.28−1.23)	0.160	
**Employment**							0.758
Unemployed	REF		REF		REF		
Employed	1.66 (0.71−3.90)	0.241	6.76 (2.10−21.75)	0.001	2.18 (1.06−4.47)	0.034	
**Anal sex days, per 10 days** ^b^	1.15 (1.08−1.23)	<0.001	1.13 (1.06−1.20)	<0.001	1.26 (1.19−1.34)	<0.001	0.168

Note: Be‐PrEP‐ared and AMPrEP studies, 17 August 2015–5 May 2018.

a. “P‐value covariable x study interaction” tests foreffect modification between covariable and study by including an interactionterm of the two (along with individual components) to the group‐based trajectory model. This is an overall test across OR comparing variable vs. low, almost daily vs. low, and always daily vs. low profiles.

b. Sum of the number of days on which anal sex occurred during follow‐up. The odds ratios can be interpreted as the increase in odds tobelong to a certain trajectory per 10‐day increase in anal sex days.

### 3.3 STIs per PrEP use trajectory

Sixty‐eight syphilis, 267 chlamydia and 290 gonorrhoea infections were diagnosed during 559.0 person‐years (PY). IR was 12.2/100PY (95% CI = 9.6−15.4) for syphilis, 47.8/100PY (95% CI = 42.4−53.9) for chlamydia and 51.9/100PY (95% CI = 46.2−58.2) for gonorrhoea. Compared to “low frequency” PrEP users, the IR of chlamydia was higher for participants with “almost daily” (IRR = 2.1, 95% CI = 1.2−3.8) and “always daily” PrEP use (IRR = 2.3, 95% CI = 1.3−4.1) (Table 3). Similarly, the IR of gonorrhoea was higher for participants with “almost daily” (IRR = 2.3, 95% CI = 1.3−4.1) and “always daily” PrEP use (IRR = 2.6, 95% CI = 1.5−4.5). Syphilis incidence did not differ between trajectories. There was no evidence that these effect sizes were different between studies (Table 3).

**Table 3:** Chlamydia, gonorrhoea and syphilis incidence stratified by PrEP use profile

	Chlamydia			Gonorrhoea			Syphilis		
	*Events/py*	*IR (95% CI)* ^a^	*aIRR* ^b^ *(95% CI)*	*Events/py*	*IR (95% CI)* ^a^	*aIRR* ^b^ *(95% CI)*	*Events/py*	*IR (95% CI)* ^a^	*aIRR* ^b^ *(95% CI)*
**Trajectory**									
Low frequency	13/57.6	22.6 (13.1−38.9)	REF	13/57.6	22.6 (13.1−38.9)	REF	5/57.6	8.7 (3.6−20.9)	REF
Variable	35/86.8	40.3 (29.0−56.2)	1.8 (1.0−3.4)	34/86.8	39.2 (28.0−54.8)	1.8 (0.9−3.4)	13/86.8	15.0 (8.7−25.8)	1.7 (0.6−4.9)
Almost daily	81/161.8	50.1 (40.3−62.3)	2.1 (1.2−3.8)	90/161.8	55.6 (45.3−68.4)	2.3 (1.3−4.1)	21/161.8	13.0 (8.5−19.9)	1.4 (0.5−3.8)
Always daily use	138/252.8	54.6 (46.2−64.5)	2.3 (1.3−4.1)	153/252.8	60.5 (51.7−70.9)	2.6 (1.5−4.5)	29/252.8	11.5 (8.0−16.5)	1.2 (0.5−3.1)
***p‐value*** ^c^ ***profile × study interaction***			0.396			0.791			0.755

**Note: Be‐PrEP‐ared and AMPrEP studies, 17 August 2015–5 May 2018**.

**Abbreviations**: aIRR, adjusted incidence rate ratio; CI, confidence interval; IR, incidence rate; py, person‐years.

a. Per 100 person‐years.

b. Adjusted for age and testing frequency per total months in follow‐up.

c. “*p*‐value profile × study interaction” tests for effect modification between profile and study by including an interaction term of the two (along with individual components) to the Poisson regression model. This is an overall test across IRR comparing variable versus low, almost daily versus low and always daily versus low profiles.

## 4 DISCUSSION

Using longitudinal daily data from 516 PrEP users in two countries, we identified four distinct trajectories of PrEP use during a median period of 73 weeks. As expected, “low frequency” PrEP use was associated with the lowest number of anal sex days and “always daily” PrEP use with the highest. Accordingly, participants using “always daily” or “almost daily” PrEP had a higher IR of chlamydia and gonorrhoea compared to “low frequency” PrEP users.

The majority of participants used PrEP on an “always daily” or “almost daily” basis. If we relate this result to the two known regimens (i.e. daily and event‐driven), these individuals were predominately daily PrEP users. “Low frequency” and “variable” PrEP use groups were mainly event‐driven PrEP users, yet 17% and 40%, respectively, of them were in fact daily PrEP users at baseline. These observations demonstrate that PrEP users have varying patterns of PrEP use and these patterns do not exactly correspond to simply “daily” or “event‐driven” PrEP. These profiles are also linked to varying degrees of sexual behaviour and risk of STI, thereby giving rise to differing PrEP needs.

There were differences in the distribution of PrEP use profiles between studies. Participants using PrEP “variable” and “almost daily” were more likely to be AMPrEP participants. It should be noted that Be‐PrEP‐ared participants were actively reminded to fill in the daily diary, contrary to AMPrEP participants, and as a result, individuals participating in Be‐PrEP‐ared were less likely to have missing daily data. We assume that missing data for weeks with incompletely filled data represent no PrEP use, and hence it becomes difficult to distinguish AMPrEP participants using PrEP “variable” or “almost daily” who truly forgot a single tablet during the week from those who forgot to record their PrEP use once during the week. Alternatively, age and certain behaviours associated with STI and HIV infection were different between studies, while these characteristics are known to influence PrEP use [10, 15]. Despite these differences, the lack of any interaction would suggest that the main results were no different across studies.

There was substantial variation in the median numbers of days per week on which PrEP was taken in participants with “low frequency” and “variable” PrEP use. Most participants with “low frequency” PrEP use had 2 or less days of PrEP use within the week. This result is somewhat surprising as the recommended schedule for event‐driven PrEP usually covers a span of at least 3 days (i.e. two pills 24–2 hours prior to a sex act, followed by a daily tablet until 48 hours after the last sex act). This level of PrEP intake would suggest inadequate adherence to the event‐driven PrEP regimen, which was uncommon in both AMPrEP [15] and Be‐PrEP‐ared [10]. Perhaps reporting 1 or 2 days of PrEP use could be from individuals who anticipated a condomless anal sex act, which eventually did not occur, and hence had no need to continue PrEP use. This hypothesis is corroborated with previous evidence from AMPrEP in which event‐driven PrEP users frequently stopped PrEP if no condomless sex occurred [15]. Taken together, the difference in PrEP use between “low frequency” and “variable” PrEP use groups could be explained by whether a condomless anal sex act followed event‐driven PrEP use.

As more information has become available about non‐daily PrEP use and the World Health Organization (WHO) and Centers for Disease Control (CDC) have recommended to include event‐driven PrEP for cis‐gender men in PrEP programmes [4, 5], commencing non‐daily PrEP regimens, or switching between regimens, may be increasingly more common. Indeed, event‐driven PrEP has increased in use within the Belgian and Dutch national PrEP programmes [17, 18] and was the preferred regimen for individuals commencing PrEP in a recent study from Western Africa [19]. Switching between regimens was also frequent in the Be‐PrEP‐ared and AMPrEP studies, with up to one‐third of participants expecting to have switched regimens at least once during follow‐up [10, 13]. However, these switches could have been partly driven by the SARS‐CoV‐2 pandemic [20, 21]. The profiles identified in our study help elucidate some of the complexity of PrEP use highlighted in these previous studies. These profiles also underline the need for clear counselling to PrEP users on how to safely initiate, discontinue and re‐start PrEP, which is not entirely evident [22, 23], in order to reduce the risks associated with incorrect use.

We found that participants with “almost daily” PrEP use were younger than participants with “low frequency” PrEP use (mean ≤2 PrEP days/week from the group‐based trajectory model). Although hypothetical, older participants might have found it easier to plan event‐driven PrEP use in accordance with their sex life [12]. Participants with “low frequency” PrEP use were more often unemployed than participants using PrEP “almost daily.” Since PrEP was provided free of charge in AMPrEP and Be‐PrEP‐ared, PrEP costs are likely not an explanation for this association. If and why unemployment plays a role in the choice of PrEP regimen should be further explored in future research.

This study has other limitations. First, some participants never or rarely used the daily diary application and were, therefore, excluded from the analysis. As certain determinants, most importantly the choice of PrEP regimen at baseline, differed between included and excluded participants, this may have induced selection bias. Second, the assumptions of imputing missing data depended on whether an individual filled in the app at least once during a given week. This method could bias trajectories towards higher levels of PrEP use if PrEP users no longer took PrEP during entire weeks without app use. Third, most study participants were white and highly educated and may not reflect the broader population using PrEP. Fourth, when Be‐PrEP‐ared and AMPrEP began enrolling participants, event‐driven PrEP was less well known and not yet included in WHO or CDC guidelines [4, 5]. This information could have led participants to favour daily regimens in both studies and thereby influenced the shape of trajectories over time. Fifth, data were obtained from demonstration projects initiated in 2015 and, although age and sexual preference are similar to those in current national PrEP programmes in the Netherlands and Belgium [17, 18], might not be representative of current practice. Lastly, data on condom use, sexual behaviour (i.e. type of sex partner) and PrEP use (i.e. number of pills taken vs. any) were assessed differently in the daily applications of Be‐PrEP‐ared and AMPrEP [10, 15, 24]. We could only base the trajectory models on any PrEP use and assess the associations with the total number of anal sex days. Data on condomless anal sex acts, which more accurately reflects the risk of HIV acquisition, and number of pills taken, which bears more importance to adherence, could have provided further insights into how PrEP was used in practice.

## 5 CONCLUSIONS

We identified four distinct PrEP use trajectories, which represented average PrEP use ranging from 2 days per week or less to daily or almost daily use. These trajectories were associated with sexual behaviour. To accommodate different schedules of PrEP use and ensure its effectiveness, it would be more appropriate to shift away from defining and presenting PrEP use as two separate regimens. We recommend to focus, rather, on how to more effectively use PrEP when the need for it changes over time. Future research should investigate how PrEP care could be adapted according to different usage patterns, for example, by evaluating whether the frequency of STI screening among participants with low levels of PrEP use can be reduced.

## COMPETING INTERESTS

The study medication for the Be‐PrEP‐ared study of Antwerp and the Amsterdam PrEP study was provided by Gilead Sciences based on unconditional grants. EH received advisory board fees from Gilead Sciences and speaker fees from Janssen‐Cilag, both paid to her institute. UD received unrestricted research grants and speaker's fees from Gilead Sciences, paid to his institute. HJCdV received grants from Medigene, and advisory board and speaker fees from Gilead Sciences, Medigene, Abbvie, Janssen‐Cilag and Willpharma, paid to his institute. MP received unrestricted research grants and speaker's fees from Gilead Sciences, Roche, Abbvie and MSD, paid to her institute. MSvdL served on an Advisory Board of MSD, paid to his institute. All other authors declare no competing interests.

## AUTHORS’ CONTRIBUTIONS

BV, TR, EH, MP, ML, MSvdL, HJCdV and UD conceptualized and designed this study and obtained funding. VWJ, AB, MSvdL, TS and TR were involved in the data analysis. VWJ, TR, AB, MSvdL, UD, HZ, LC, HZ, MvdE, HJCdV, CK, IDB, TS, BV, MP, ML and EH were involved with the interpretation of the data. VWJ drafted the manuscript. All authors read and approved the final manuscript.

## FUNDING

The Tale of Two Cities project was funded by ZonMW (grant number: 522008009). TR is a postdoctoral fellow of the Research Foundation—Flanders. The Be‐PrEP‐ared study was funded by the Applied Biomedical Research (TBM) Program of the Belgium Research Agency (IWT). CT/NG diagnostic kits for Real Time PCR used during the Be‐PrEP‐ared study were donated by Abbott. The AMPrEP project received funding as part of the H‐Team Initiative from ZonMw (grant number 522002003), the National Institute for Public Health and the Environment and GGD research funds. The H‐Team initiative is being supported by the Aidsfonds Netherlands (grant number 2013169), Stichting Amsterdam Diner Foundation, Gilead Sciences Europe Ltd (grant number PA‐HIV‐PREP‐16‐0024), Gilead Sciences (protocol numbers CO‐NL‐276‐4222, CO‐US‐276‐1712), Janssen Pharmaceuticals (reference number PHNL/JAN/0714/0005b/1912fde), M.A.C AIDS Fund, and ViiV Healthcare (PO numbers 3000268822, 3000747780).

## Supporting information

Supporting Information

## Data Availability

The AMPrEP data are owned by the Public Health Service of Amsterdam; the Be‐PrEP‐ared data by the Institute of Tropical Medicine. Original data can be requested by submitting a study proposal to the steering committee of AMPrEP or Be‐PrEP‐ared. The pooled dataset can be requested by submitting a study proposal to the corresponding author. Request for further information can also be submitted to the corresponding author. The AMPrEP and Be‐PrEP‐ared steering committees verify each proposal for compatibility with general objectives, ethical approval and informed consent forms of the AMPrEP and Be‐PrEP‐ared studies, and potential overlap with ongoing studies. There are no restrictions to obtaining the data and all data requests will be processed in a similar way.
