# Supplementary material for: Trajectories of PrEP use among men who have sex with men: a pooled analysis of two prospective, observational cohort studies
Source: J Int AIDS Soc. 2023 Jul 27;26(7):e26133. doi: 10.1002/jia2.26133 (PMC10374882; doi:10.1002/jia2.26133)
Supplement: Supplementary file 1 — Supporting Information [file JIA2-26-e26133-s001.docx]

**SUPPLEMENT TO:**

**Trajectories of PrEP use among men who have sex with men: a pooled analysis of two prospective, observational cohort studies**

Vita W. Jongen, Thijs Reyniers, Maarten Schim van der Loeff, Tom Smekens, Elske Hoornenborg, Mark van den Elshout, Hanne Zimmermann, Liza Coyer, Chris Kenyon, Irith de Baetselier, Udi Davidovich, Henry J. C. de Vries, Maria Prins, Marie Laga, Bea Vuylsteke, Anders Boyd

**TABLE OF CONTENTS:**

[SUPPLEMENTARY TABLES 2](#_Toc126586907)

[Supplementary Table 1. Model fit statistics for selecting the number of groups in the group based trajectory model of PrEP use per week^1^ 2](#_Toc126586908)

[Supplementary Table 2: Socio-demographic and sexual behavior characteristics at PrEP initiation of PrEP users who used the applications ≥10% (n=520) and <10% (n=56) during the AMPrEP (Amsterdam, The Netherlands) and Be-PrEP-ared (Antwerp, Belgium) cohort study periods, August 2015 to December 2016. 3](#_Toc126586909)

[Supplementary Table 3: Comparison of socio-demographic and sexual behavior characteristics at PrEP initiation between individuals included in the AMPrEP (Amsterdam, The Netherlands) versus Be-PrEP-ared (Antwerp, Belgium) cohort study, August 2015 to December 2016. 5](#_Toc126586910)

[Supplementary Table 4. Univariable determinants of PrEP use profiles. Be-PrEP-ared and AMPrEP studies, 17 August 2015 to 5 May 2018. 7](#_Toc126586911)

[SUPPLEMENARY FIGURES 9](#_Toc126586912)

[Supplementary Figure 1. Trajectories of PrEP use per week over time among MSM and transgender PrEP users, stratified on the Be-PrEPared (A) and AMPrEP (B) studies, 17 August 2015 to 5 May 2018. 9](#_Toc126586913)

# SUPPLEMENTARY TABLES

## Supplementary Table 1. Model fit statistics for selecting the number of groups in the group based trajectory model of PrEP use per week^1^

| No. of groups | BIC (N=516)^2^ | BIC (N=33,011)^3^ | AIC | Entropy |
| --- | --- | --- | --- | --- |
| 2 | -32,635.12 | -32,655.92 | -32,613.89 | 0.979 |
| 3 | -31,200.73 | -31,231.92 | -31,231.92 | 0.965 |
| 4 | -30,581.99 | -30,623.58 | -30,539.53 | 0.956 |
| 5 | -30,080.77 | -30,028.78 | -29,975.71 | 0.937 |
| 6 | -29,723.70 | -29,786.08 | -29,660.01 | 0.907 |
| 7 | -29,572.72 | -29,499.95 | -29,425.64 | 0.927 |
| 8 | -29,315.60 | -29,398.77 | -29,230.67 | 0.916 |

**Abbreviations:** AIC, Akaike information criterion; BIC, Bayesian information criterion; No., number; PrEP, pre-exposure prophylaxis

1. We selected the number of groups based on the highest entropy (above 0.900), the lowest BIC and all trajectories having a marginal prevalence of >10%
2. 516 indicates the number of participants included in the model
3. 33,011 indicates the number of non-missing observations included in the model

## Supplementary Table 2: Socio-demographic and sexual behavior characteristics at PrEP initiation of PrEP users who used the applications ≥10% (n=516) and <10% (n=55) during the AMPrEP (Amsterdam, The Netherlands) and Be-PrEP-ared (Antwerp, Belgium) cohort study periods, August 2015 to December 2016.

|  | **≥10% app use** | | **<10% app use** | |  |
| --- | --- | --- | --- | --- | --- |
|  | **(n=516)** | | **(n=55)** | | *p-value* |
|  | *n^1^* | *%^1^* | *n^1^* | *%^1^* |  |
| **Study location** |  |  |  |  | *<0.001* |
| Antwerp | 194 | 38% | 3 | 5% |  |
| Amsterdam | 322 | 62% | 52 | 95% |  |
| **Choice of PrEP regimen** |  |  |  |  | *0.002* |
| Daily | 392 | 76% | 31 | 56% |  |
| Event-driven | 124 | 24% | 24 | 44% |  |
| **Age (years)** |  |  |  |  |  |
| Median [IQR] | 39 | [32-47] | 39 | [33-52] | *0.207* |
| <35 year | 171 | 33% | 18 | 33% | *0.332* |
| 35 - 44 year | 184 | 36% | 15 | 27% |  |
| ≥45 year | 161 | 31% | 22 | 40% |  |
| **Self-declared racial-ethnic background** |  |  |  |  | *0.511* |
| White | 448 | 87% | 46 | 84% |  |
| Non-white | 68 | 13% | 9 | 16% |  |
| **Highest education level** |  |  |  |  | *0.062* |
| No college/university | 111 | 22% | 18 | 33% |  |
| College/university | 402 | 78% | 37 | 67% |  |
| **Employment** |  |  |  |  | *0.005* |
| Employed | 438 | 86% | 39 | 71% |  |
| Unemployed | 74 | 14% | 16 | 29% |  |
| **Steady relationship** |  |  |  |  | *0.918* |
| No | 283 | 55% | 30 | 55% |  |
| Yes | 229 | 45% | 25 | 45% |  |
| **Living situation** |  |  |  |  | *0.903* |
| Alone | 265 | 51% | 30 | 55% |  |
| With partner | 170 | 33% | 17 | 31% |  |
| With others | 81 | 16% | 8 | 15% |  |
| **Sexual identity^2^** |  |  |  |  | *0.058* |
| Exclusively homosexual | 484 | 94% | 48 | 87% |  |
| Not exclusively homosexual | 31 | 6% | 7 | 13% |  |
| **CAS with casual partner^3^** |  |  |  |  | *0.354* |
| No | 23 | 4% | 1 | 2% |  |
| Yes | 493 | 96% | 54 | 98% |  |
| **Post-exposure prophylaxis used^3^** | |  |  |  | *0.036* |
| No | 461 | 89% | 54 | 98% |  |
| Yes | 55 | 11% | 1 | 2% |  |
| **Sexually transmitted infection^3,4^** | |  |  |  | *<0.001* |
| No | 274 | 53% | 46 | 84% |  |
| Yes | 242 | 47% | 9 | 16% |  |

**Abbreviations**: CAS, condomless anal sex; IQR, interquartile range; PEP, post-exposure prophylaxis; PrEP, pre-exposure prophylaxis

We used rank sum tests for continuous variables and Pearson’s χ2 or Fisher’s exact tests for categorical variables. Data were missing for education (n=3), employment (n=4), steady relationship (n=4), sexual identity (n=1)

1. Unless stated otherwise
2. Sexual identity was measures on a 7-point Likert scale ranging from 1=exclusively heterosexual to 7=exclusively homosexual. In order to pool the AMPrEP and Be-PrEP-ared data correctly exclusively (7 on the Likert scale) and mainly homosexual (6 on the Likert scale) were combined to “exclusively homosexual”. Of note, in previous AMPrEP papers, mainly homosexual was categorized as “not exclusively homosexual”.
3. In the 6 months before baseline
4. At least one bacterial sexually transmitted infection (i.e. syphilis, urethral or rectal chlamydia or gonorrhea)

## Supplementary Table 3: Comparison of socio-demographic and sexual behavior characteristics at PrEP initiation between participants included in the analyses of the AMPrEP (Amsterdam, The Netherlands) versus Be-PrEP-ared (Antwerp, Belgium) cohort study, August 2015 to December 2016.

|  | **Be-PrEP-ared** | | **AMPrEP** | |  |
| --- | --- | --- | --- | --- | --- |
|  | **(n=194)** | | **(n=322)** | | *p-value* |
|  | *n^1^* | *%^1^* | *n^1^* | *%^1^* |  |
| **Number of days completed in the app^2^** |  |  |  |  |  |
| Median [IQR] | 511 | [508-511] | 494 | [348-510] | *<0.001* |
| **Choice of PrEP regimen** |  |  |  |  | *0.730* |
| Daily | 45 | 23% | 79 | 25% |  |
| Event-driven | 149 | 77% | 243 | 75% |  |
| **Age (years)** |  |  |  |  |  |
| Median [IQR] | 39 | [32-44] | 40 | [32-47] | *0.349* |
| <35 year | 62 | 32% | 109 | 34% | *0.002* |
| 35 - 44 year | 86 | 44% | 98 | 30% |  |
| ≥45 year | 46 | 24% | 115 | 36% |  |
| **Self-declared racial-ethnic background** |  |  |  |  | *0.338* |
| White | 172 | 89% | 276 | 86% |  |
| Non-white | 22 | 11% | 46 | 14% |  |
| **Highest education level** |  |  |  |  | *0.510* |
| No college/university | 39 | 20% | 72 | 23% |  |
| College/university | 155 | 80% | 247 | 77% |  |
| **Employment** |  |  |  |  | *0.992* |
| Employed | 166 | 86% | 272 | 86% |  |
| Unemployed | 28 | 14% | 46 | 14% |  |
| **Steady relationship** |  |  |  |  | *0.683* |
| No | 105 | 54% | 178 | 56% |  |
| Yes | 89 | 46% | 140 | 44% |  |
| **Living situation** |  |  |  |  | *0.596* |
| Alone | 95 | 49% | 170 | 53% |  |
| With partner | 65 | 34% | 105 | 33% |  |
| With others | 34 | 18% | 47 | 15% |  |
| **Sexual identity^3^** |  |  |  |  | *0.011* |
| Exclusively homosexual | 189 | 97% | 295 | 92% |  |
| Not exclusively homosexual | 5 | 3% | 26 | 8% |  |
| **CAS with casual partner^4^** |  |  |  |  | *0.468* |
| No | 7 | 4% | 16 | 5% |  |
| Yes | 187 | 96% | 306 | 95% |  |
| **Post-exposure prophylaxis used^4^** | |  |  |  | *0.014* |
| No | 165 | 85% | 296 | 92% |  |
| Yes | 29 | 15% | 26 | 8% |  |
| **Sexually transmitted infection^4,5^** | |  |  |  | *<0.001* |
| No | 78 | 40% | 196 | 61% |  |
| Yes | 116 | 60% | 126 | 39% |  |
| **Anal sex days^6^** |  |  |  |  |  |
| Median [IQR] | 136 | [87-204] | 109 | [59-160] | *<0.001* |

**Abbreviations**: CAS, condomless anal sex; IQR, interquartile range; PEP, post-exposure prophylaxis; PrEP, pre-exposure prophylaxis

We used rank sum tests for continuous variables and Pearson’s χ2 or Fisher’s exact tests for categorical variables. Data were missing for education (n=3), employment (n=4), steady relationship (n=4), sexual identity (n=1)

1. Unless stated otherwise
2. Participants could complete a maximum of 511 days in each of the applications
3. Sexual identity was measures on a 7-point Likert scale ranging from 1=exclusively heterosexual to 7=exclusively homosexual. In order to pool the AMPrEP and Be-PrEP-ared data correctly exclusively (7 on the Likert scale) and mainly homosexual (6 on the Likert scale) were combined to “exclusively homosexual”. Of note, in previous AMPrEP papers, mainly homosexual was categorized as “not exclusively homosexual”.
4. In the 6 months before baseline
5. At least one bacterial sexually transmitted infection (i.e. syphilis, urethral or rectal chlamydia or gonorrhea)
6. Sum of the number of days on which anal sex occurred during follow-up

## Supplementary Table 4. Univariable determinants of PrEP use profiles. Be-PrEP-ared and AMPrEP studies, 17 August 2015 to 5 May 2018.

|  | **Profile** | | | | | | |
| --- | --- | --- | --- | --- | --- | --- | --- |
|  | **Variable vs. Low** | | **Almost daily vs. Low** | | **Always daily vs. Low** | | |
|  | *OR (95% CI)* | *p-value* | *OR (95% CI)* | *p-value* | *OR (95% CI)* | *p-value* | |
| **Study site** |  |  |  |  |  |  | |
| Antwerp | REF |  | REF |  | REF |  | |
| Amsterdam | 2.02 (0.97-4.21) | 0.059 | 1.83 (0.99-3.36) | 0.054 | 1.05 (0.62-1.78) | 0.864 | |
| **Age** |  |  |  |  |  |  | |
| <35 years | REF |  | REF |  | REF |  | |
| 35-44 years | 0.97 (0.42-2.27) | 0.354 | 0.67 (0.33-1.37) | 0.276 | 1.15 (0.60-2.18) | 0.672 | |
| ≥45 years | 1.09 (0.47-2.53) | 0.836 | 0.61 (0.29-1.25) | 0.176 | 1.02 (0.53-1.97) | 0.942 | |
| **Self-declared racial-ethnic background** |  |  |  |  |  |  | |
| White | REF |  | REF |  | REF |  | |
| Non-white | 1.07 (0.40-2.82) | 0.896 | 1.05 (0.44-2.53) | 0.910 | 0.91 (0.39-2.13) | 0.823 | |
| **Highest education level** |  |  |  |  |  |  | |
| No college/university | REF |  | REF |  | REF |  | |
| College/university | 2.18 (0.93-5.11) | 0.072 | 1.23 (0.59-2.56) | 0.587 | 1.37 (0.76-2.45) | 0.294 | |
| **Employment** |  |  |  |  |  |  | |
| Unemployed | REF |  | REF |  | REF |  | |
| Employed | 1.95 (0.82-4.60) | 0.129 | 3.27 (1.45-7.33) | 0.004 | 2.73 (1.43-5.22) | 0.002 | |
| **Steady relationship** |  |  |  |  |  |  | |
| No | REF |  | REF |  | REF |  | |
| Yes | 1.11 (0.48-1.63) | 0.697 | 1.02 (0.50-2.08) | 0.958 | 1.09 (0.69-1.73) | 0.717 | |
| **Living situation** |  |  |  |  |  |  | |
| Alone | REF |  | REF |  | REF |  | |
| With partner | 1.13 (0.54-2.34) | 0.749 | 0.48 (0.22-1.05) | 0.065 | 1.26 (0.72-2.22) | 0.419 | |
| With others | 1.01 (0.36-2.83) | 0.984 | 0.89 (0.35-2.24) | 0.798 | 1.27 (0.59-2.72) | 0.542 | |
| **Sexual identity** |  |  |  |  |  |  | |
| Exclusively homosexual | REF |  | REF |  | REF |  | |
| Not exclusively homosexual | 2.75 (0.28-27.22) | 0.387 | 0.99 (0.23-4.28) | 0.988 | 0.44 (0.13-1.51) | 0.190 | |
| **CAS with casual partner^1^** |  |  |  |  |  |  | |
| No | REF |  | REF |  | REF |  |  |
| Yes | 2.84 (0.52-15.67) | 0.230 | 1.08 (0.35-3.36) | 0.892 | 2.48 (0.72-8.59) | 0.151 |  |
| **Post-exposure prophylaxis used^1^** |  |  |  |  |  |  |  |
| No | REF |  | REF |  | REF |  |  |
| Yes | 1.42 (0.46-4.34) | 0.541 | 1.40 (0.53-3.72) | 0.501 | 1.10 (0.44-2.73) | 0.837 |  |
| **Sexually transmitted infection^1,2^** |  |  |  |  |  |  |  |
| No | REF |  | REF |  | REF |  |  |
| Yes | 1.32 (0.66-2.65) | 0.435 | 1.24 (0.68-2.25) | 0.485 | 1.34 (0.79-2.30) | 0.279 |  |
| **Anal sex days, per 10 days^3^** | 1.14 (1.07-1.22) | <0.001 | 1.16 (1.09-1.23) | <0.001 | 1.29 (1.21-1.37) | <0.001 |  |

**Abbreviations:** CAS, condomless anal sex; CI, confidence interval; OR, odds ratio; PEP, post-exposure prophylaxis

Data missing for: employment (n=4), sexual identity (n=1), steady relationship (n=4)

1. In the 6 months before baseline
2. At least one bacterial sexually transmitted infection (i.e., syphilis, or urethral or rectal chlamydia or gonorrhea) at baseline.
3. Sum of the number of days on which anal sex occurred during follow-up. The odds ratios can be interpreted as the increase in odds to belong to a certain trajectory per 10-day increase in anal sex days

# SUPPLEMENARY FIGURES

## Supplementary Figure 1. Trajectories of PrEP use per week over time among MSM and transgender PrEP users, stratified on the Be-PrEPared (A) and AMPrEP (B) studies, 17 August 2015 to 5 May 2018.

| **A** | 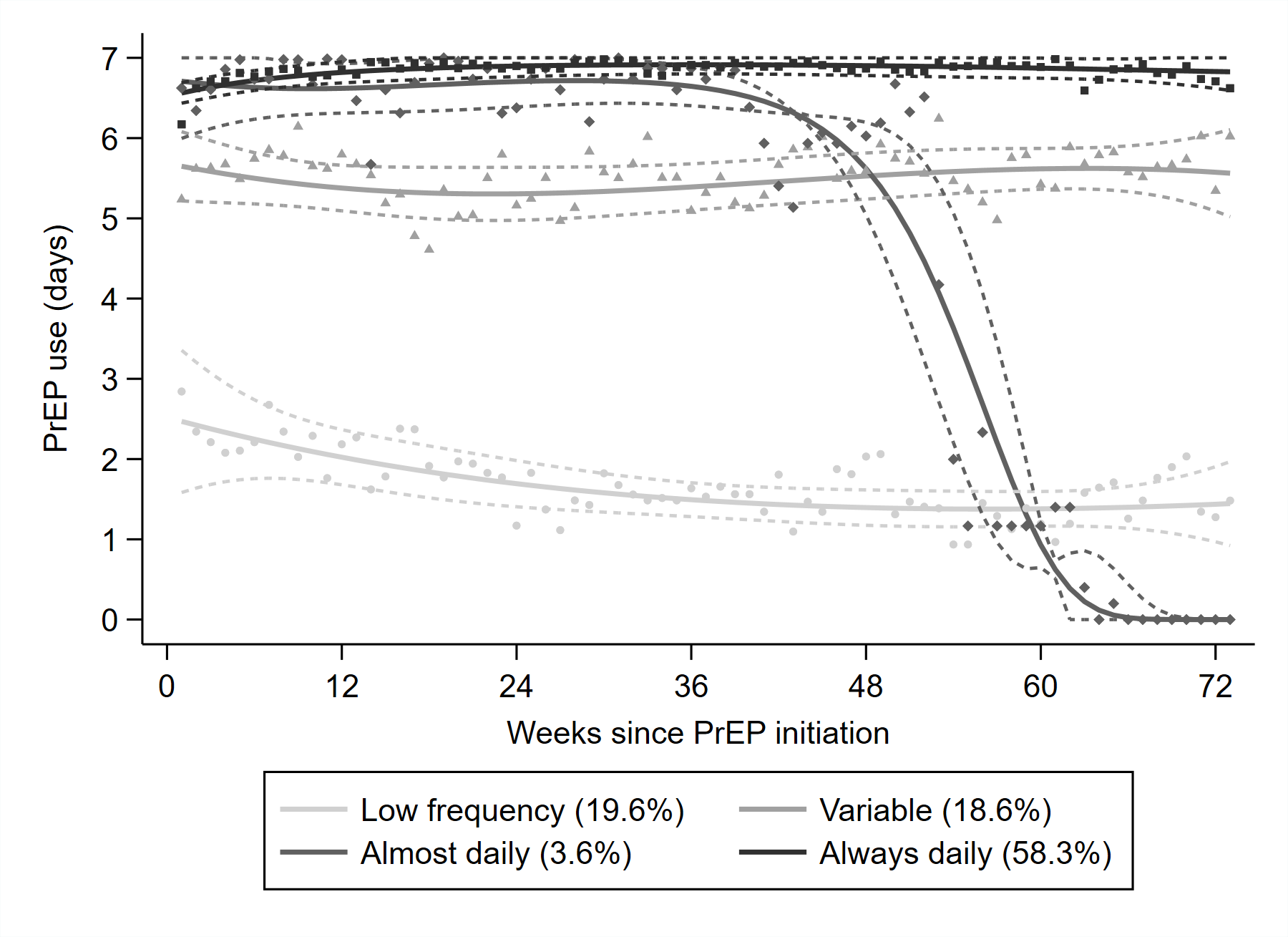 |
| --- | --- |
| **B** | 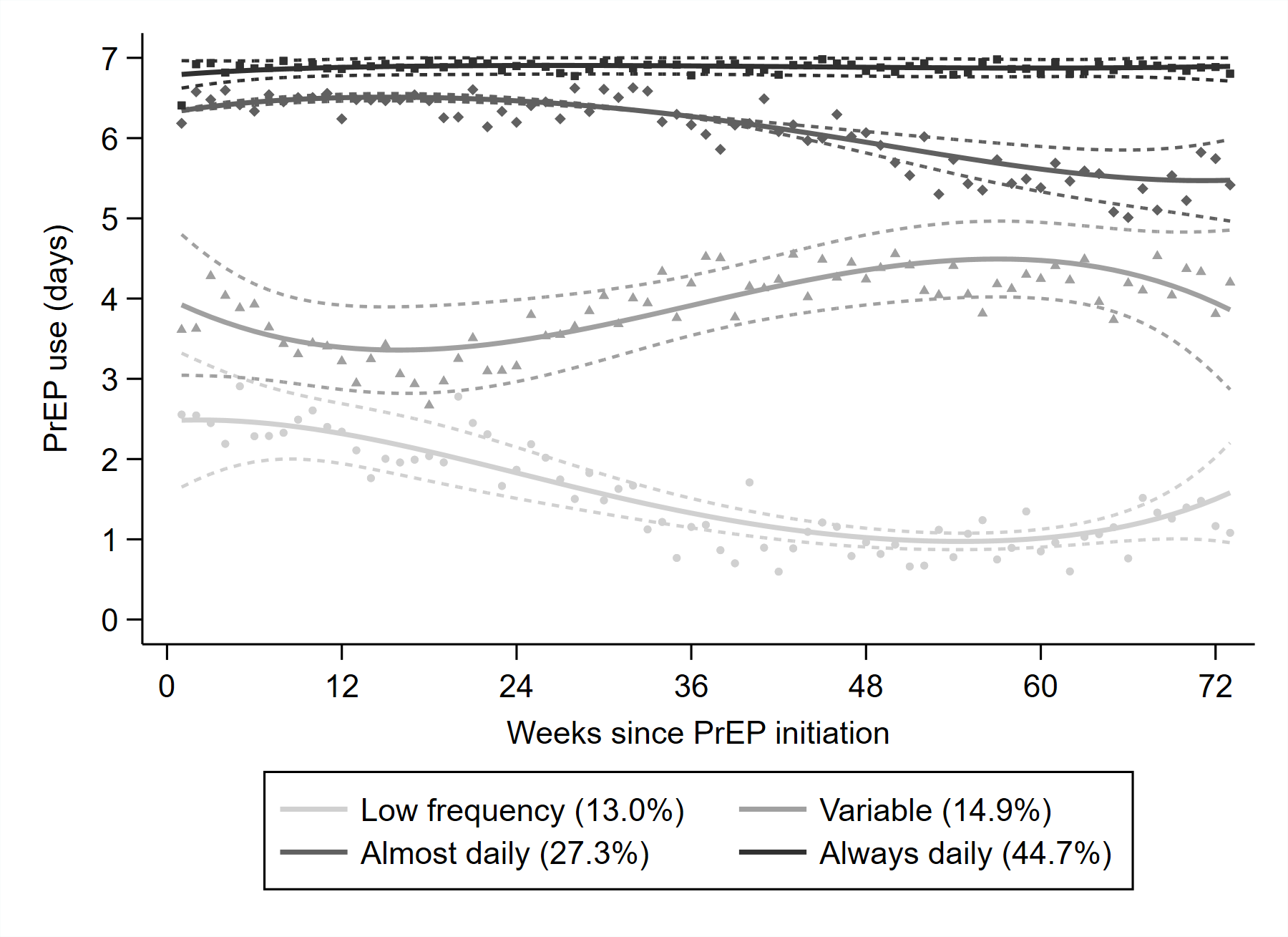 |

*Plotted symbols indicate the observed group mean number of tablets per week within each trajectory; the plotted lines indicate the trajectory;* *dashed lines indicate the 95% confidence intervals of the trajectory.*
